# A computational model of the stellate cell microcircuit in the auditory brainstem

**DOI:** 10.1186/1471-2202-15-S1-P153

**Published:** 2014-07-21

**Authors:** Timothy Esler, David B Grayden

**Affiliations:** 1 NeuroEngineering Laboratory, Dept. of Electrical & Electronic Engineering, University of Melbourne, Victoria 3010, Australia; 2 Centre for Neural Engineering, University of Melbourne, Victoria 3010, Australia

## 

The cochlear nucleus (CN) is the first processing center of auditory signals from the cochlea. Several distinct neuronal circuits have been identified that form different parallel representations of auditory information from auditory nerve fibers (ANFs). One cell circuit of interest is the stellate cell microcircuit of the ventral cochlear nucleus (VCN), which is centered on two populations of stellate cells, known as T and D stellate cells [1]. The output of the T stellate (TS) cell population has been shown experimentally to accurately encode important frequency components over a wide range of input sound pressure levels (SPL) and in the presence of significant signal noise [2]. It is thought that one purpose of the stellate cell circuit is to increase the dynamic range and the signal-to-noise ratio (SNR) of the incoming signal at all levels in order to more accurately highlight the most significant frequencies. A clear application of this in speech processing is the identification of vowels, which requires detection of the first two or three formant frequencies.

A neural network model of the stellate microcircuit was created using leaky integrate-and-fire neurons for each cell in the network. The key mechanisms incorporated to achieve SNR and dynamic range improvement were wide-band lateral inhibition and selective processing of excitatory and inhibitory inputs [3]. The number and type of cell inputs, the network connectivity, and the time constants associated with cellular dynamics were made, where possible, to agree with experimental observations. In addition, the performance of the model in response to a variety of stimuli was adjusted to achieve the desired signal processing functions regarding noise reduction, peak identification and dynamic range.

To quantify the enhancement achieved by the network, two signal-to-noise metrics were used. SNR metric 1 measures the level of noise reduction and is calculated as the ratio of the average output rate of cells within 1.5 critical bands of the known stimulus frequency to the average background output rate. SNR metric 2 measures the level of peak enhancement and is calculated as the ratio of the maximum output rate of the cells within 1.5 critical bands of the known stimulus frequency to the maximum background output rate. The metrics were calculated for the input signal, the input ANF population, and the TS cell population. Each metric was calculated for the first three formants of the vowel /o:/ and for a range of input SNR values. The SNR metric 1 was significantly higher than for the ANF population for the majority of input SNR levels. Similarly, SNR metric 2 was also higher for the TS cell population than for the ANF population. This demonstrates that the TS cells improved the ability to decode the spectral information in noise and the model illustrates the mechanisms by which this is achieved.
